# Implementation teledermatologischer Konsile am Universitätsklinikum Leipzig

**DOI:** 10.1007/s00105-024-05463-z

**Published:** 2025-01-18

**Authors:** Susanne Triebswetter, Jan C. Simon, Anna-Theresa Seitz

**Affiliations:** Klinik und Poliklinik für Dermatologie, Venerologie und Allergologie, Universitätsklinikum Leipzig AöR, Philipp-Rosenthal-Str. 23, 04103 Leipzig, Deutschland

**Keywords:** Store-and-forward, Telemedizin, eHealth, Stationär, Dermatologische Begutachtung, Store-and-forward, Telemedicine, eHealth, Inpatient, Dermatological expert opinion

## Abstract

**Hintergrund:**

Zahlreiche Patient:innen, die in eine Klinik stationär aufgenommen werden, weisen nebenbefundlich Hautveränderungen auf. Am Universitätsklinikum Leipzig AöR wurden in einem Jahr ca. 800 Konsile durch die Klinik und Poliklinik für Dermatologie, Venerologie und Allergologie beantwortet.

**Ziel der Arbeit:**

Es erfolgten die Implementation einer teledermatologischen Konsillösung und anschließend die retrospektive Evaluation dieser und der daraus resultierenden Zufriedenheit von konsilstellenden und konsilbeantwortenden Ärzt:innen.

**Material und Methoden:**

Es erfolgte eine Auswertung aller Konsilanfragen, die mittels Store-and-Forward-Technologie über die Fotodokumentations-App imitoCAM®-App (imito AG Zürich/Schweiz) und das Krankenhausinformationssystem SAP® (SAP Deutschland SE & Co. KG Walldorf/Deutschland) zwischen 01.02.2023 und 31.07.2023 beantwortet wurden, sowie von Fragebögen zur Zufriedenheit, die nach erfolgtem Konsil den beteiligten Ärzt:innen zugeschickt wurden.

**Ergebnisse:**

In die Auswertung gingen 419 Konsile ein, davon wurden 90 teledermatologisch beantwortet. Die Konsilärzt:innen gaben an, dass in 92 % der Fälle eine rein telemedizinische Behandlung erfolgen konnte, und 90,9 % der konsilstellenden Ärzt:innen beurteilten die technische Umsetzung als gut bis sehr gut, und 47,5 % der Konsilärzt:innen gaben eine Zeitersparnis von mindestens 30–60 min im Vergleich zur Beantwortung von Bett‑/Ambulanzkonsilen an.

**Diskussion:**

Unsere Untersuchung zeigt das enorme Potenzial der Teledermatologie auch im stationären Setting. Die Implementation von Telekonsilen ermöglicht eine effizientere Konsilbeantwortung, reduziert zeit- und kostenintensive Transporte in die Hautklinikambulanz und bietet Patient:innen eine komfortablere dermatologische Begutachtung mit vermindertem Infektionsrisiko.

**Graphic abstract:**

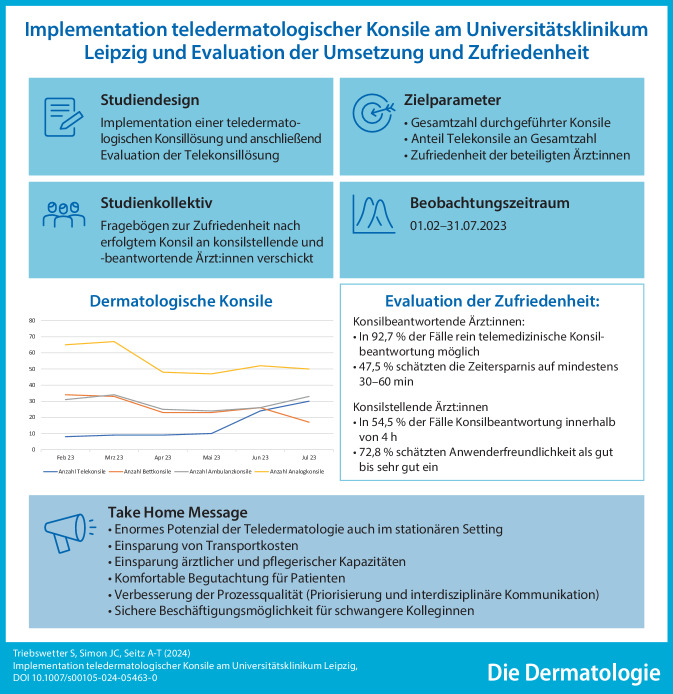

## Abstract (en)

**Background:**

Numerous patients admitted to a general hospital present skin changes as secondary findings. Approximately 800 dermatological consultations are performed annually at the Leipzig University Medical Center.

**Objectives:**

The aim of this study was, after implementation of teledermatological consulting, the retrospective evaluation of that and the resulting satisfaction of the physicians involved.

**Materials and methods:**

We carried out an analysis of all consultation requests, which were answered using store-and-forward technology via the photo documentation app imitoCAM® (imito AG Zurich/Switzerland) and the clinical information system SAP® (SAP Deutschland SE & Co. KG Walldorf/Germany) from 1 February to 31 July 2023, as well as of questionnaires on satisfaction, which were sent to the participating physicians after the consultation.

**Results:**

Our analysis included 419 consultation requests, 90 of which were answered teledermatologically. The consultation dermatologists stated that 92% of the cases could be solved using only telemedical instruments. Moreover, 90.9% of the consulting physicians rated the technical implementation as good to very good and 47.5% of the consultation dermatologists estimated a time saving of at least 30–60 min.

**Conclusions:**

Our study shows the enormous potential of teledermatology in the inpatient setting. Implementation of teleconsultation enables more efficient consultations, reduces time- and cost-intensive transportation and offers patients a more comfortable dermatological evaluation with reduced risk of infection.

Viele Patient:innen, die in eine Klinik stationär aufgenommen werden, weisen nebenbefundlich Hautveränderungen auf. Um Transportkosten und ärztliche sowie pflegerische Kapazitäten einzusparen und Patient:innen eine komfortablere Begutachtung zu ermöglichen, implementierten wir am Universitätsklinikum Leipzig AöR eine teledermatologische Konsillösung. In diesem Beitrag stellen wir die Ergebnisse einer Evaluation der Telekonsillösung und der daraus resultierenden Zufriedenheit von konsilstellenden und konsilbeantwortenden Ärzt:innen vor.

## Hintergrund

Am Universitätsklinikum Leipzig werden pro Jahr etwa 800 dermatologische Konsile durchgeführt. Die Leipziger Hautklinik ist ein solitär stehendes Gebäude. Dies ist für viele interne Prozesse von Vorteil. Bei dermatologischen Fragestellungen werden die Patient:innen der anderen Abteilungen jedoch entweder per Transport in die Ambulanz der Hautklinik gebracht oder Fach- und Oberärzt:innen visitieren die Patient:innen auf den jeweiligen Stationen. Zwischen den einzelnen Kliniken bestehen zum Teil Distanzen von bis zu 1,4 km. Im Rahmen der SARS-CoV-2(„severe acute respiratory syndrome coronavirus type 2“)-Pandemie sollten im Frühjahr 2020 interne Patiententransporte sowie Konsile auf anderen Stationen zur Vermeidung von Infektionen so weit wie möglich reduziert werden. Vor diesem Hintergrund und der vorher bestehenden kosten- und zeitintensiven Konsilbeantwortung wurde eine telemedizinische Lösung angestrebt.

Als visuell geprägtes Fach ist in der Dermatologie die Bearbeitung einer Vielzahl von Konsilanfragen anhand klinischer Angaben und aktueller Patientenfotos möglich. Gemäß der deutschen Leitlinie zur Praxis der Teledermatologie von Augustin et al. aus dem Jahr 2018 wird Telemedizin in der Kommunikation und Befundbesprechung unter Ärzt:innen ein hoher Nutzen zugeschrieben [1]. Mehrere Übersichtsarbeiten sind zu dem Schluss gekommen, dass – unter Berücksichtigung der Einschränkungen sowie der Beherrschung der notwendigen technischen Fähigkeiten – die Teledermatologie für viele Indikationen eine wertvolle Bereicherung darstellen kann [2–4]. Die Beantwortung von dermatologischen Konsilanfragen anhand von klinischen Angaben und Patientenfotos bietet folgende Vorteile für Patient:innen und Behandler:innen:

- Einsparung von Transportkosten,
- Einsparung ärztlicher und pflegerischer Kapazitäten,
- komfortable Begutachtung für Patient:innen,
- Verbesserung der Prozessqualität (Priorisierung und interdisziplinäre Kommunikation),
- sichere Beschäftigungsmöglichkeit für schwangere Kolleginnen.

Der Leitfaden der deutschsprachigen Dermatologen zur Praxis der Teledermatologie empfiehlt daher, den Einsatz einer teledermatologischen Behandlung „immer dann in Erwägung zu ziehen, wenn relevante Zusatznutzen für die Patienten ohne relevante Nachteile für sie und für die Versorgenden zu erwarten sind“ [3].

Ziel dieser retrospektiven Arbeit war nach Implementation einer teledermatologischen Konsillösung die Evaluation dieser und der daraus resultierenden Zufriedenheit von konsilstellenden und konsilbeantwortenden Ärzt:innen.

## Patienten und Methodik

### Konsilstellung

**Abb. 1:**
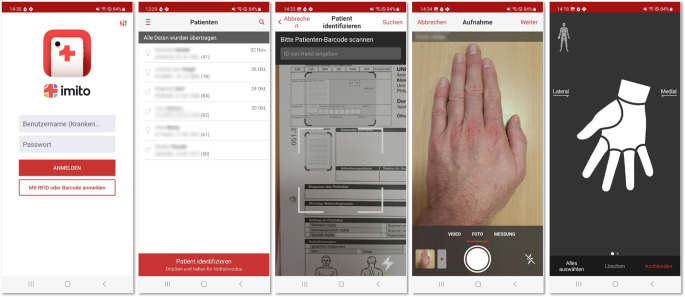
Beispielhafter Ablauf der Fotodokumentation mittels imitoCAM-App® (imito AG Zürich/Schweiz) (Einloggen mittels Benutzernamen und Passwort, Patientauswahl bzw. Einscannen des Patientbarcodes, Erstellen eines Fotos mittels Handy- oder Tabletkamera und Zuordnung der entsprechenden Körperstelle auf einem Piktogramm)

**Abb. 2:**
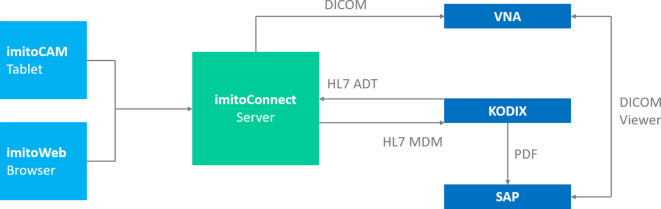
Schematische Übersicht über den Ablauf der Datenübermittlung von der Fotodokumentations-App imitoCAM-App® an das Krankenhausinformationssystem SAP® (SAP Deutschland SE & Co. KG Walldorf/Deutschland). *DICOM* Digital Imaging and Communications in Medicine, *HL7* Health Level 7, *ADT* Admission Discharge Transfer, *MDM* Medical Document Management, *VNA* Vendor Neutral Archive

Die teledermatologischen Konsile erfolgten mittels Store-and-Forward-Technologie, d. h. die Konsilanfrage inklusive Bilddokumentation wurden gespeichert und dann zeitversetzt begutachtet und beantwortet. Dies ist mit dem klinischen Alltag besser vereinbar als ein Echtzeitkonsil in Form einer Videosprechstunde. Die Fotoaufnahmen werden durch die Anwendung eines mobilen Endgerätes (z. B. Handy oder Tablet) mittels imitoCAM-App (imito AG Zürich/Schweiz) aufgenommen und der Patientenakte über imitoWeb (als Webservice) hinzugefügt (Abb. 1). Sowohl die App als auch der Webservice der Fotodokumentations-App erhalten einen HL7(Health Level 7)-ADT(„admission discharge transfer“)-Datensatz aus den zur klinischen Dokumentation und Administration am Universitätsklinikum Leipzig verwendeten SAP®-Komponenten IS‑H und i.s.h.med (SAP Deutschland SE & Co. KG Walldorf/Deutschland). Damit werden die Aufnahmedatensätze von Patient:innen nach Neuaufnahme bzw. bestehende Aufnahmedatensätze direkt in der App und im Webservice verfügbar gemacht. Patient:innen können dadurch in der App/im Webservice gesucht werden, und die aufgenommenen Fotoaufnahmen können direkt im Fallkontext zugeordnet werden. Nach Abschluss der Fotodokumentation in der App oder im Webservice werden die Bilder zum einem als DICOM(Digital Imaging and Communications in Medicine)-Nachricht an das am Klinikum eingesetzte VNA („vendor neutral archive“) gesendet und dort der Patientenhistorie unter Kennzeichnung des jeweiligen Falls zugeordnet (Abb. 2). Zum anderen werden die Bilder als eingebettetes pdf-Dokument mit zusätzlichen Informationen über die Körperregion der Aufnahme und Kommentaren zur Bildstudie via HL7 MDM (Medical Document Management) an das Krankenhausinformationssystem SAP® i.s.h.med versendet und stehen dort ebenfalls fallbezogen im Patientenprofil zur Einsicht zur Verfügung. Die Behandelnden haben somit die Möglichkeit, in dem von ihnen verwendeten Dokumentationssystem sowie in der Viewing-Komponente des VNA die Bilddaten fallbezogen und unmittelbar nach Aufnahme und Abschluss der Bildstudie einzusehen. Daten werden in der Fotodokumentations-App pseudonymisiert und verschlüsselt übertragen und gespeichert. Ausschließlich befugte Personen erhalten Zugriff auf die Fotodokumentations-App sowie auf das klinikinterne Krankenhausinformationssystem mittels persönlicher Zugangsdaten.

Anschließend wird für die jeweiligen Patient:innen ein klinischer Auftrag für ein dermatologisches Konsil im Krankenhausinformationssystem ausgelöst, der zusätzliche Angaben zu Anamnese, Verlauf des klinischen Befunds und Fragestellung des Konsils enthält. Nach Absenden des Auftrags erscheint dieser in einer Worklist der dermatologischen Ambulanz.

Die zuständigen Konsilärzt:innen können die Anfrage inklusive Fotodokumentation einsehen und ihre Empfehlungen eintragen. Anschließend wird der klinische Auftrag abgeschlossen, und es erscheint ein Konsilbefund im Krankenhausinformationssystem.

### Auswertung zur Zufriedenheit

**Abb. 3:**
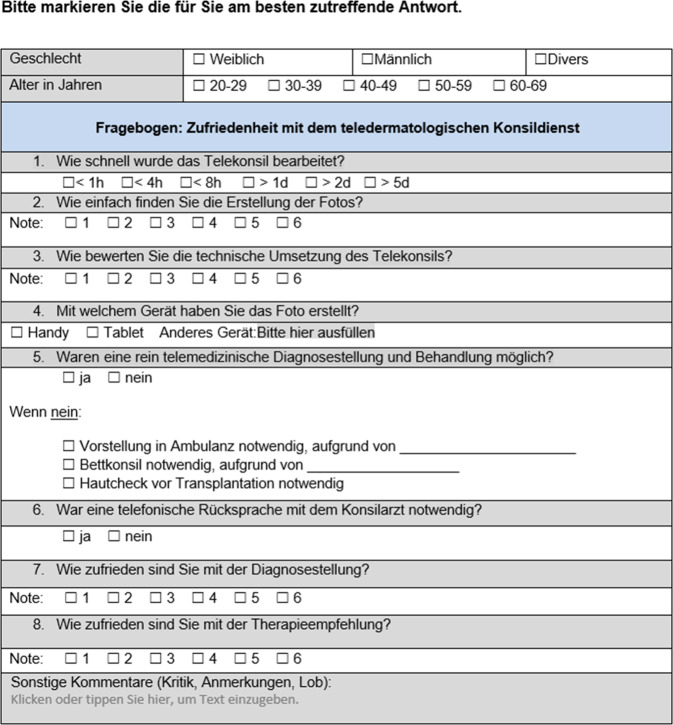
Muster des Fragebogens, der nach Beantwortung eines teledermatologischen Konsils an die konsilstellenden Ärzt:innen geschickt wurde (Copyright Anna-Theresa Seitz)

Nach Abschluss eines teledermatologischen Konsils wurde den jeweiligen konsilstellenden und konsilbeantwortenden Ärzt:innen via klinikinterner E‑Mail ein Link zu strukturierten Fragebögen (Abb. 3 und 4) zugeschickt. Die Beantwortung erfolgte anonym über evasys® (Version 9.1, evasys GmbH Lüneburg/Deutschland), eine Datenschutz-konforme webbasierte Software für die Automatisierung von Befragungen. Die Fragebögen enthielten u. a. Fragen zu Geschlecht, Alter und Ausbildungstand der jeweiligen Ärzt:innen, zu Wartezeit auf die Konsilbeantwortung bzw. Zeitersparnis für die konsilbeantwortenden Ärzt:innen sowie Nachfragen, ob eine rein telemedizinische Beantwortung möglich oder ob eine Rücksprache nötig war, sowie Fragen zu Anwenderfreundlichkeit, technischer Umsetzung und Zufriedenheit mit Diagnosestellung und Therapieempfehlung. Die Befragung erfolgte über 6 Monate im Zeitraum vom 01.02.2023 bis 31.07.2023. Zusätzlich wurden folgende weitere Aspekte in die Auswertung mit einbezogen:

- Gesamtzahl der dermatologischen Konsile im Untersuchungszeitraum, aufgeteilt nach Konsilart (Telekonsil, Ambulanzkonsil, Bettkonsil),
- Entwicklung der Anzahl teledermatologischer Konsile über die Beobachtungsdauer,
- Art der Diagnose(n).

**Abb. 4:**
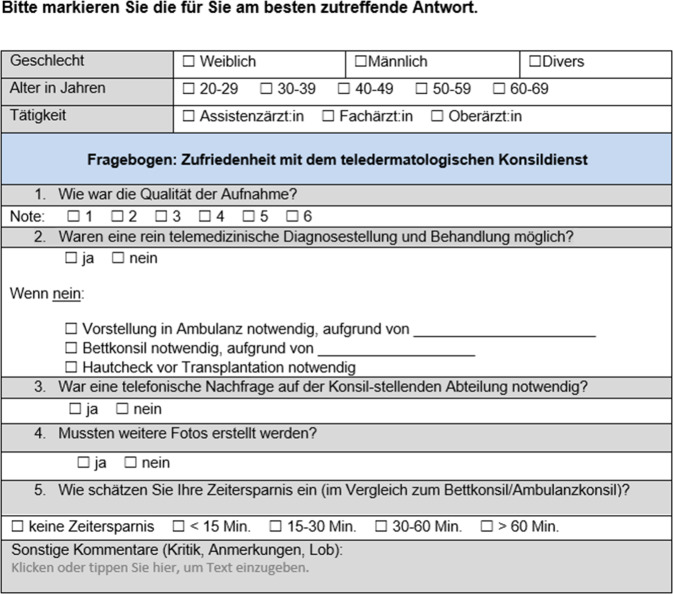
Muster des Fragebogens, der nach Beantwortung eines teledermatologischen Konsils an die Konsilärzt:innen geschickt wurde (Copyright Anna-Theresa Seitz)

Die Datenauswertung erfolgte am 10.08.2023. Zu diesem Zeitpunkt hatten 11 konsilstellende Ärzt:innen und 41 konsilbeantwortende Ärzt:innen den ihnen zugeschickten Fragebogen ausgefüllt. Die Datenerhebung und -auswertung erfolgten statistisch-deskriptiv via evasys und Microsoft Excel (Microsoft Office Professional Plus 2016, Microsoft Corporation Redmond Washington/USA).

## Ergebnisse

**Abb. 5:**
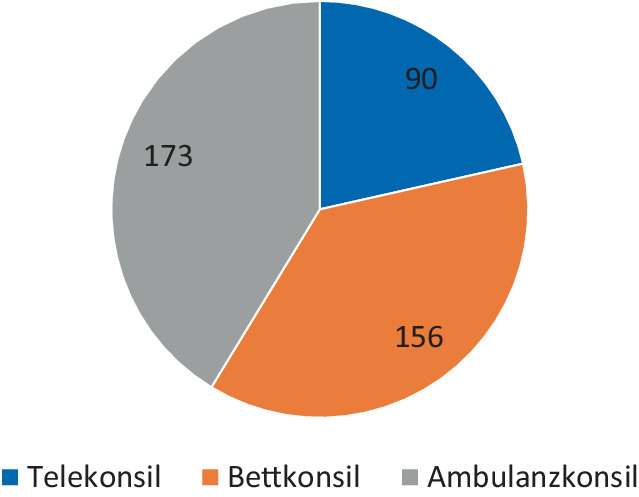
Anzahl der vom 01.02. bis 31.07.2023 durchgeführten dermatologischen Konsile, aufgeteilt nach Konsiltyp

**Abb. 6:**
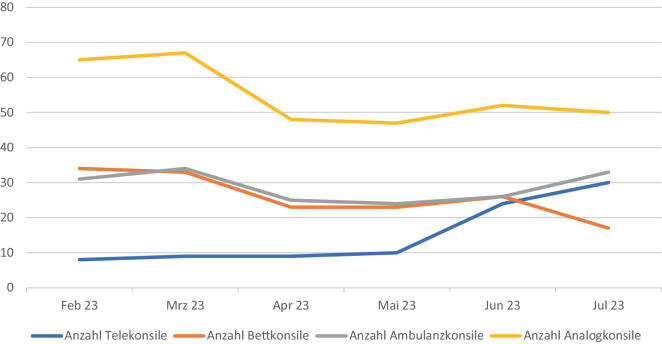
Anzahl der vom 01.02. bis 31.07.2023 durchgeführten dermatologischen Konsile, aufgeteilt nach Konsilart und verteilt auf die einzelnen Monate (wobei Bett- und Ambulanzkonsile zusätzlich als Analogkonsile zusammengefasst wurden)

Im untersuchten Zeitraum (01.02.–31.07.2023) wurden am Universitätsklinikum Leipzig insgesamt 419 dermatologische Konsile durchgeführt, davon 21,5 % Telekonsile, 41,3 % Ambulanzkonsile und 37,2 % Bettkonsile (Abb. 5). Im Lauf des Beobachtungszeitraums wurden zunehmend mehr Konsilanfragen telemedizinisch beantwortet. Während im Februar 2023 nur 8 Konsilanfragen teledermatologisch beantwortet wurden, konnten im Juli 2023 bereits 30 dermatologische Konsile telemedizinisch gelöst werden (Abb. 6). Im Rahmen der teledermatologischen Konsilbeantwortung wurden 49 verschiedene Diagnosen gestellt. Die häufigsten Konsilanfragen bezogen sich auf die folgenden Diagnosen: Arzneimittelexanthem (7 Fälle), malignes Melanom (6 Fälle), Candida-Intertrigo (6 Fälle), Stauungsdermatitis (4 Fälle) und Ulcus cruris (3 Fälle).

### Fragebogen zur Zufriedenheit der konsilbeantwortenden Ärzt:innen

Von 90 erfolgten teledermatologischen Konsilen im Untersuchungszeitraum füllten 41 Konsilärzt:innen den ihnen zugesandten Fragebogen aus (Antwortrate von 45,6 %). Die Konsilärzt:innen waren in der Mehrzahl weiblich (78 %) und in der Altersgruppe zwischen 30 und 39 (61 %). Die Qualität der Bildaufnahme schätzten 77,5 % als gut bis sehr gut ein. Nur in 5 % der Fälle mussten weitere Fotos erstellt werden. Eine rein telemedizinische Diagnosestellung und Behandlung waren in 92,7 % der Fälle möglich, jedoch war in 53,7 % der Konsile noch eine telefonische Rücksprache mit den konsilstellenden Kolleg:innen notwendig. Bei den restlichen 7,3 % der Konsile, die nicht rein teledermatologisch gelöst werden konnten, war in der Hälfte der Fälle ein Bettkonsil, in der anderen Hälfte der Fälle ein Ambulanzkonsil z. B. zur Entnahme einer Probeexzision notwendig. Insgesamt gab die Mehrheit der Dermatolog:innen eine deutliche Zeitersparnis im Vergleich zu Bett‑/Ambulanzkonsilen an. Knapp die Hälfte schätzte die Zeitersparnis auf mindestens 30–60 min.

### Fragebogen zur Zufriedenheit der konsilstellenden Ärzt:innen

Von 90 erfolgten teledermatologischen Konsilen im Untersuchungszeitraum füllten 11 der konsilstellenden Ärzt:innen den ihnen zugesandten Fragebogen aus (Antwortrate von 12,2 %). Die Ärzt:innen waren in der Mehrzahl männlich (54,5 %) und in der Altersgruppe zwischen 20 und 29 (45,5 %). Es gaben 54,5 % der Ärzt:innen an, dass ihre Konsilanfrage innerhalb von 4 h beantwortet wurde. Nahezu 75 % der Konsile wurden innerhalb eines Tages beantwortet. In 72,7 % der Fälle waren eine rein telemedizinische Diagnosestellung und Behandlung möglich, jedoch war bei gut der Hälfte der Konsile (54,5 %) eine telefonische Rücksprache mit dem/der Konsilärzt:in notwendig. Dies war in Übereinstimmung mit den Angaben der konsilbeantwortenden Ärzt:innen. Bei den Konsilen, die nicht rein teledermatologisch gelöst werden konnten, war in einem Drittel der Fälle eine Hautkrebsvorsorge vor Organtransplantation notwendig, die übrigen zwei Drittel erforderten ein Ambulanzkonsil z. B. aufgrund eines operativen Vorgehens. Die Bilder wurden in allen Fällen mittels Tablet erstellt. Die Anwenderfreundlichkeit des Systems/Gerätes bei Erstellung der Fotodokumentation schätzten 72,8 %, die technische Umsetzung des Telekonsils sogar 90,9 % der konsilstellenden Ärzt:innen als gut bis sehr gut ein. Mit Diagnosestellung und Therapieempfehlung zeigten sich knapp 82 % zufrieden bis sehr zufrieden.

## Diskussion

Telemedizinische Anwendungen haben sich in den letzten Jahren, nicht zuletzt auch aufgrund der SARS-CoV-2-Pandemie, deutlich weiterentwickelt und verbreitet. Dieser Entwicklung wurde auf dem Feld der Dermatologie bereits durch die Erstellung einer Sk2-Leitlinie sowie eines Leitfadens für Teledermatologie Rechnung getragen [1, 3]. Dort wird aus der aktuellen Datenlage die Schlussfolgerung gezogen, dass „telemedizinische Unterstützung der dermatologischen Behandlung und Prävention bei Einsatz leistungsfähiger Systeme, Kenntnis ihrer Anwendung sowie Beachtung der Indikationen und Kontraindikationen einen erheblichen Mehrnutzen darstellt“ [3]. Die Implementation der telemedizinischen Beantwortung von dermatologischen Konsilen mithilfe einer Fotodokumentations-App stellt sowohl für die betroffenen Patient:innen als auch die beteiligten Ärzt:innen einen Mehrnutzen dar.

Knapp 73 % der konsilstellenden Ärzt:innen stuften die Anwenderfreundlichkeit des Systems/Gerätes bei Erstellung der Fotodokumentation als gut bis sehr gut ein. Die technische Umsetzung des Telekonsils wurde sogar von 90,9 % für gut bis sehr gut befunden. Diese Ergebnisse zeigen, dass durch die Fotodokumentations-App eine unkomplizierte Befunddokumentation von Hauterscheinungen möglich ist, was wiederum sowohl die Durchführung von Telekonsilen als auch die interdisziplinäre Kommunikation und Zusammenarbeit z. B. im Rahmen eines Tumorboards erleichtert. Darüber hinaus kann durch diese Technik eine sichere Übermittlung von Daten an den Hintergrunddienst erfolgen, was eine Verbesserung von Behandlungsqualität und Priorisierung zur Folge hat.

Knapp die Hälfte der Dermatolog:innen gab an, mindestens eine Zeitersparnis von 30–60 min gehabt zu haben. Es wurden 72,7 % der Konsile noch am gleichen Tag bearbeitet. Durch die Implementation von Telekonsilen konnten Konsilärzt:innen zeitintensive Wege vermeiden. Dies ermöglichte eine schnellere Bearbeitung der Konsilanfragen, die Einsparung kostenintensiver Transporte in die Ambulanz der Hautklinik und eine Reduktion des Infektionspotenzials für Patient:innen und Ärzt:innen.

Als Limitation dieser Arbeit ist die geringe Rücklaufquote bei den konsilstellenden Ärztinnen zu nennen. Dennoch zeigt sich über den Beobachtungszeitraum eine deutliche Zunahme der Zahl der Telekonsile, was wir als zunehmende Zufriedenheit mit dem Konzept der teledermatologischen Konsilbeantwortung werten. Eine weitere Limitation besteht darin, dass kein Patient:innen-Feedback erhoben wurde. Dies wird Inhalt einer zukünftigen Studie sein. Eine Einschränkung des Konzepts der dermatologischen Telekonsile an sich ist die Tatsache, dass nicht alle Fragestellungen mittels Fotodokumentation lösbar sind. Dies betrifft insbesondere Fragestellungen, die eine Probeexzision oder eine dermatoskopische Untersuchung erforderlich machen.

Telemedizin kann nicht komplett den Arzt-Patienten-Kontakt ersetzen. In manchen Fällen ist zur Untersuchung, Diagnosestellung und Besprechung des Krankheitsbildes und Erklärung therapeutischer und präventiver Maßnahmen ein direkter Arzt-Patienten-Kontakt notwendig.

Im Leitfaden für Teledermatologie wird empfohlen, den Einsatz einer teledermatologischen Behandlung „immer dann in Erwägung zu ziehen, wenn relevante Zusatznutzen für die Patienten ohne relevante Nachteile für sie und für die Versorgenden zu erwarten sind“. Die Entscheidung zur ausschließlich teledermatologischen Durchführung des Konsils muss von Fall zu Fall getroffen werden und ist abhängig vom jeweiligen Krankheitsbild. Die verlässlichste Studienlage liegt für Psoriasis, Ekzemerkrankungen und chronische Wunden vor. Wenn jedoch eine entsprechende Selektion nach Krankheitsbild vorgenommen wird, deutet die aktuelle Studienlage darauf hin, dass die Ergebnisse, die über telemedizinische Anwendungen erreicht wurden, mit einer Präsenzvorstellung vergleichbar sind [3].

Durch die Implementation dermatologischer Telekonsile in unserer Klinik konnten wir eine schnellere und effizientere Konsilbeantwortung ermöglichen und so die Zufriedenheit der beteiligten Kolleg:innen steigern. Die betroffenen Patient:innen erhielten unkompliziert und zeitnah eine Diagnose sowie eine therapeutische Empfehlung, ohne lange Transportwege und Wartezeiten in Kauf nehmen zu müssen.

## Fazit für die Praxis

- Bereits in einem kurzen Beobachtungszeitraum von 6 Monaten konnten wir eine deutliche Zunahme und hohe Akzeptanz der telemedizinisch gelösten dermatologischen Konsile beobachten.
- Darüber hinaus lässt sich die Technik selbst auch für zahlreiche andere Zwecke einsetzen, wie z. B. die Operationsvorbereitung oder die klinisch-histologische Korrelation.
- Eine weitere interne sowie auch externe Ausweitung ist denkbar.

## Data Availability

Daten, die die Ergebnisse dieser wissenschaftlichen Publikation stützen, sind auf begründete Anfrage bei der korrespondierenden Autorin erhältlich.

## References

[1] Augustin M (2020) 013-097l_S2k_Teledermatologie, S 2021–2003

[2] Elsner P (2020) Teledermatology in the times of COVID-19—a systematic review. J Dtsch Dermatol Ges 18:841–845. 10.1111/ddg.14180 33448667 10.1111/ddg.14180

[3] Augustin M, Wimmer J, Biedermann T, Blaga R, Dierks C, Djamei V et al (2018) Praxis der Teledermatologie. J Dtsch Dermatol Ges 5:6–57. 10.1111/ddg.13512 10.1111/ddg.13512 29998512

[4] Trettel A, Eissing L, Augustin M (2018) Telemedicine in dermatology: findings and experiences worldwide—a systematic literature review. J Eur Acad Dermatol Venereol 32:215–224. 10.1111/jdv.14341 28516492 10.1111/jdv.14341

